# Therapeutic Potential of Traditional Oriental Medicines in Targeting Tau Pathology: Insights from Cell-free and Cell-based Screening

**DOI:** 10.2174/0109298673295901240311072440

**Published:** 2024-03-13

**Authors:** Hyun Ha Park, Byeong-Hyeon Kim, Seol Hwa Leem, Yong Ho Park, Hyunju Chung, Doo-Han Yoo, Insu Park, Yunkwon Nam, Sujin Kim, Soo Jung Shin, Minho Moon

**Affiliations:** 1 Department of Biochemistry, College of Medicine, Konyang University, 158, Gwanjeodong-ro, Seo-gu, Daejeon, 35365, Republic of Korea;; 2 Department of Core Research Laboratory, Medical Science Research Institute, Kyung Hee University Hospital Gangdong, Seoul, 05278, Republic of Korea;; 3 Research Institute for Dementia Science, Konyang University, 158, Gwanjeodong-ro Seo-gu, Daejeon, 35365, Republic of Korea;; 4 Department of Occupational Therapy, Konyang University, 158, Gwanjeodong-ro, Seo-gu, Daejeon, 35365, Republic of Korea;; 5 Department of Biomedical Engineering, Konyang University, 158, Gwanjeodong-ro, Seo-gu, Daejeon, 35365, Republic of Korea

**Keywords:** Neurodegenerative disease, dementia, Alzheimer’s disease, tau, traditional oriental medicines, screening assay

## Abstract

**Background:**

Traditional Oriental Medicines (TOMs) formulated using a variety of medicinal plants have a low risk of side effects. In previous studies, five TOMs, namely *Dangguijakyaksan*, *Hwanglyeonhaedoktang*, *Ukgansan*, *Palmijihwanghwan*, and *Jowiseungchungtang* have been commonly used to treat patients with Alzheimer’s disease (AD). However, only a few studies have investigated the effects of these five TOMs on tau pathology.

**Objective:**

This study aimed to examine the effect of five TOMs on various tau pathologies, including post-translational modifications, aggregation and deposition, tau-induced neurotoxicity, and tau-induced neuroinflammation.

**Methods:**

Immunocytochemistry was used to investigate the hyperphosphorylation of tau induced by okadaic acid. In addition, the thioflavin T assay was used to assess the effects of the TOMs on the inhibition of tau K18 aggregation and the dissociation of tau K18 aggregates. Moreover, a water-soluble tetrazolium-1 assay and a quantitative reverse transcription polymerase chain reaction were used to evaluate the effects of the TOMs on tau-induced neurotoxicity and inflammatory cytokines in HT22 and BV2 cells, respectively.

**Results:**

The five TOMs investigated in this study significantly reduced okadaic acid-induced tau hyperphosphorylation. *Hwanglyeonhaedoktang* inhibited the aggregation of tau and promoted the dissociation of tau aggregates. *Dangguijakyaksan* and *Hwanglyeonhaedoktang* attenuated tau-induced neurotoxicity in HT22 cells. In addition, *Dangguijakyaksan*, *Hwanglyeonhaedoktang*, *Ukgansan*, and *Palmijihwanghwan* reduced tau-induced pro-inflammatory cytokine levels in BV2 cells.

**Conclusion:**

Our results suggest that five TOMs are potential therapeutic candidates for tau pathology. In particular, *Hwanglyeonhaedoktang* showed the greatest efficacy among the five TOMs in cell-free and cell-based screening approaches. These findings suggest that *Hwanglyeonhaedoktang* is suitable for treating AD patients with tau pathology.

## 1 INTRODUCTION

Tauopathies are neurodegenerative disorders characterized by abnormal tau inclusions, such as Alzheimer's disease (AD), frontotemporal dementia with parkinsonism-17 (FTDP-17), and corticobasal degeneration (CBD) [1]. AD, the most common cause of dementia, poses a global health challenge [2]. AD is characterized by irreversible, progressive cognitive dysfunction. Its neuropathological hallmarks are the presence of extracellular plaques containing amyloid beta (Aβ) peptides [3] and intracellular neurofibrillary tangles (NFTs) formed by hyperphosphorylated, insoluble, and filamentous tau proteins. In particular, NFTs are closely related to neuronal cell death [4]. In the early pathological stages, tau becomes hyperphosphorylated and dissociates from microtubules, altering cytoskeleton dynamics [5]. Moreover, abnormally hyperphosphorylated tau interacts with normal tau instead of tubulin, resulting in the development of tau oligomers. These tau oligomers are then mislocated to the somatodendritic compartment [6], where they undergo further hyperphosphorylation and conformational changes to form an insoluble β-sheet structure. These oligomeric species promote the development of paired helical filaments (PHF) and NFTs. In particular, tau aggregate toxicity directly causes neurodegeneration, such as synaptic or neuronal loss [7-9], and induces neuroinflammation, such as pro-inflammatory responses and microgliosis [10-13]. Similarly, the distribution and extent of NFTs are positively correlated with brain atrophy [14-16]. Moreover, tau accumulation has been highly correlated with cognitive decline in clinical studies [17-19]. Therefore, a tau-targeting approach is essential for the treatment of tauopathies, including AD [20].

Various therapeutic approaches targeting tau have been proposed [21]. Tau pathology-targeted approaches include 1) inhibition of tau expression [22], 2) inhibition of tau aggregation [23], 3) immunization with tau [24], 4) regulation of tau degradation [25], and 5) stabilization of microtubules [26]. Currently, two tau kinase inhibitors, Fasudil and LY3372689, are in phase 2 clinical trials [27, 28]. In addition, TRx0237 (LMTX), which can inhibit tau aggregation, is in a phase 3 clinical trial [29]. Furthermore, clinical trials are currently being conducted to investigate the efficacy of gosuranemab, tilavonemab, and semorinemab, all of which can reduce the pathological forms of tau [30, 31]. Despite numerous tau-targeting strategies, anti-tau therapeutics have not yet progressed beyond the early phases of clinical trials. Therefore, the present study aimed to identify novel substances that can target various tau pathologies.

Traditional oriental medicines (TOMs) are herbal medicines formulated from various medicinal plants; they are taken over a long period of time, have a low risk of side effects, and are proposed as candidates for treating neurodegenerative diseases, including AD [32, 33]. Indeed, the Yangxue Qingnao pill, a Chinese TOM, is currently in a phase 2 clinical trial (NCT04780399) because of its proven beneficial effects on learning and memory in animal models of AD [34, 35]. With the increasing interest in TOMs related to AD, some studies have investigated the therapeutic effect of AD and the mechanisms of the bioactive substances found in medicinal plants and the herbal formulas used in TOMs [32, 36, 37]. TOMs may exert synergistic effects by acting multifunctionally on multiple targets, such as Aβ pathology, tau pathology, and neuroinflammation in neurodegenerative diseases, including AD [38-43]. Previous studies have demonstrated five TOMs that are commonly used in the clinical treatment of AD patients with cognitive impairment, namely Dangguijakyaksan (DJS), Hwanglyeonhaedoktang (HLT), Ukgansan (UGS), Palmijihwanghwan (PJH), and Jowiseungchungtang (JWS) [32, 44]. Interestingly, it has been known that among these five TOMs, some are related to the alleviatory effect of Aβ pathology and Aβ-induced neuroinflammation in AD [45, 46]. In particular, the attenuative effects of the components of the five TOMs on tau pathology are widely recognized. Wogonin (WG), a component of HLT, decreased the level of hyperphosphorylated-tau and improved cognitive function in 3xTg mice, an AD animal model of AD [47]. Moreover, *Uncaria rhynchophylla* (UR), a component of UGS, inhibited Aβ-induced tau phosphorylation and regulated tau aggregation/dissociation [36, 48]. Furthermore, *Panax ginseng* from JWS inhibited tau hyperphosphorylation and ameliorated cognitive dysfunction in the D-galactose-induced AD animal model [49]. These results suggest that TOMs containing a variety of components can alleviate tau pathology, indicating the potential of TOMs as promising therapeutic agents for AD targeting various tau pathologies.

Although some studies have demonstrated the alleviating effects of TOMs on the hyperphosphorylation of tau [36], there has been no systematic analysis of the effects of the commonly used five TOMs on various tau pathologies. Therefore, we aimed to clearly investigate the effects of these five TOMs on various tau pathologies. In particular, we elucidated the effects of the TOMs using tau pathology-based cell-free and cell-based screening strategies. We used the thioflavin T (ThT) assay as a fluorescent probe of beta-sheet-rich structures for the examination of anti- and dis-aggregation of tau. Moreover, we utilized cell-based analyses to investigate hyperphosphorylated tau, tau-induced neurotoxicity, and tau-induced inflammatory responses.

## 2 MATERIALS AND METHODS

### 2.1 Chemicals and Materials

ThT was purchased from the Tokyo Chemical Industry (Tokyo, Japan). Dulbecco's phosphate-buffered saline (DPBS) was obtained from Welgene (Gyeongsan, Republic of Korea). Heparin sodium salt from the porcine intestinal mucosa and methylene blue (MB) were purchased from Sigma-Aldrich (St. Louis, MO, USA). DJS, HLT, PJH, and UGS were purchased from Tsumura (Tokyo, Japan). DJS is composed of six dried medicinal herbs: 4.0 g of peony root, 4.0 g of *Atractylodes lancea* rhizome (ALR), 4.0 g of *Alisma* rhizome (AR), 4.0 g of *Poria* sclerotium (PS), 3.0 g of *Cnidium* rhizome (CR), and 3.0 g of Japanese *Angelica* root (JAR). HLT is prepared from the extract of a mixture of dried plants: 1.5 g of *Scutellaria* root, 1.0 g of *Coptis* rhizome, 1.0 g of *Gardenia* fruit, and 0.75 g of *Phellodendron* bark. PJH is a mixture of eight dried medicinal herbs: 3.0 g of *Rehmannia* root, 1.5 g of *Cornus* fruit, 1.5 g of *Dioscorea* rhizome, 1.5 g of AR, 1.5 g of PS, 1.25 g of moutan bark, 0.5 g of cinnamon bark, and 0.25 g of Powdered Processed *Aconite* root. UGS is composed of seven crude drugs: 4.0 g of ALR, 4.0 g of PS, 3.0 g of CR, 3.0 g of *Uncaria* hook, 3.0 g of JAR, 2.0 g of *Bupleurum* root, and 1.5 g of *Glycyrrhiza*. The JWS herbal formula was obtained from Kyung Hee University Korean Medicine Hospital (Gangdong-gu, Seoul, Korea). JWS is prepared from the extract of a mixture of dried plants: 8.0 g of *Coicis semen*, 8.0 g of *Castaneae semen*, 6.0 g of raphani semen, 6.0 g of longanae arillus, 4.0 g of liriopis tuber, 4.0 g of Platycodi radix, 4.0 g of *Acori gramineri* rhizoma, 4.0 g of Thujae semen, 4.0 g of zizyphi semen, 4.0 g of massa medicata fermentata, 3.0 g of ephedrae herba, 3.0 g of *Schisandrae fructus*, 3.0 g of amomi semen, and 3.0 g of polygalae radix.

### 2.2 Expression and Purification of Recombinant Tau K18

This study used a recombinant K18 fragment with four repeats of a critical binding domain linked to tau aggregation, which aggregates faster than full-length tau [50, 51]. The 129-residue-long tau K18 fragment was expressed and purified in *Escherichia coli (E. coli)* BL21 (DE3) cells after being cloned from full-length human tau (hTau40). Tau K18 was commercially purified by KeyProgen (Daejeon, Republic of Korea). 6H-BcohTau was transformed into *E. coli* BL21(DE3) cells for protein expression. Pre-culture was grown in LB medium in a shaking incubator at 37°C until the OD at 600 nm reached 0.6, and then the gene was induced with 1 mM (IPTG) at 20°C overnight. The cells were centrifuged at 6,000 rpm for 10 min. The supernatant was removed, and the pellet was washed two times with buffer (50 mM Tris-HCl, 1 mM EDTA, pH 8.0). The samples were centrifuged at 6,000 rpm for 10 min. The cell pellet was resuspended from resuspension buffer (50 mM tris-HCL, 0.2 M NaCl). Sonication helped the final lysis of the pellet and centrifuged at 12,000 rpm for 30 min. The purity of the proteins was confirmed by 20% sodium dodecyl sulfate-polyacrylamide gel electrophoresis (Fig. **S1**). The Tau K18 amino acid sequence was as follows: QTAPVPMPDL KNVKSKIGST ENLKHQPGGG KVQIINKKLD LSNVQSKCGS KDNIKHVPGG GSVQIVYKPV DLSKVTSKCG SLGNIHHKPG GGQVEVKSEK LDFKDRVQSK IGSLDNITHV PGGGNKKIE.

### 2.3 Cell Lines and Culture Conditions

HT22 mouse hippocampal neuronal and BV2 mouse microglial cells were provided by Dr. Hyunju Chung of Kyung Hee University Hospital Gangdong. HT22 mouse hippocampal neuronal cells were cultured in Dulbecco’s modified Eagle’s Medium (DMEM) (WelGENE), which comprised 10% fetal bovine serum (WelGENE) and 100 units/mL penicillin-streptomycin (Gibco, Waltham, MA, USA). The cells were incubated in a humidified 5% CO_2_ atmosphere at 37°C. BV2 cells were cultured in Dulbecco’s modified Eagle’s Medium/F12 Nutrient Mixture Ham (DMEM/F12) (WelGENE), which comprised 10% fetal bovine serum (WelGENE) and 100 units/mL penicillin-streptomycin (Gibco), and incubated in a humidified 5% CO_2_ atmosphere at 37°C.

### 2.4 Immunocytochemistry (ICC)

HT22 cells were seeded on slides in a removable chamber (ibidi GmbH, Munich, Germany) at a density of 1.5 × 10^4^ cells and cultured for 24 h. The cells were treated with five TOMs in the presence of 40 nM okadaic acid and incubated for 4 h. After 4 h, the cells were fixed in 4% paraformaldehyde solution for 10 min and permeabilized with 0.5% Triton X-100 in phosphate-buffered saline (PBS) for 15 min. The cells were then blocked in a solution of 5% bovine serum albumin (BSA) in PBS for 1 h and finally, were incubated with anti-phospho-tau (T231) antibody (1:1,000; Thermo Fisher Scientific Inc., MA, USA) at 37°C for 2 h. After washing three times with PBS, the cells were incubated with fluorescence-conjugated secondary antibodies, donkey Alexa 488-conjugated anti-rabbit IgG (1:1,000; Thermo Fisher Scientific Inc.), and prepared in PBS containing 0.01% Tween 20 and 3% BSA for 1 h. Next, the cells were washed three times with PBS. Finally, the cells were mounted on slides containing 4,6-diamidino-2-phenylindole.

### 2.5 Evaluation of the Effect of the Five TOMs on Tau K18 Aggregation

To investigate the anti-aggregation effects of the five TOMs on tau, a ThT assay was performed using tau K18. First, the tau K18 stock solution was diluted to a final concentration of 35 μM by adding DPBS. To induce tau aggregation, 0.1 mg/mL heparin was added along with tau K18 to black polypropylene 96-well plates. Next, the plates were treated with various concentrations of the five TOMs at 10, 100, and 1,000 μg/mL and 100 μM MB (positive control) [52]. The treated plates were then incubated at 37°C for 21 h. Next, the ThT solutions were added to the plates and incubated for an additional 3 h. ThT fluorescence intensities were measured using a SpectraMax iD3 Multi-Mode Microplate Reader (Molecular Devices, CA, USA) with excitation and emission wavelengths of 440 nm and 484 nm, respectively.

### 2.6 Evaluation of the Effect of the Five TOMs on the Dissociation of Tau K18 Aggregates

ThT assay was used to determine the effects of the five TOMs on the dissociation of preformed tau aggregates. To induce tau aggregation, 35 μM tau K18 was incubated with 0.1 mg/mL heparin at 37°C for 24 h. Different concentrations of the five TOMs at 10, 100, and 1,000 μg/mL were then added to the incubated mixture, and the incubation was continued for an additional 21 h. Subsequently, ThT was applied to the 96-well plate containing the mixture at a concentration of 15 μM in 50 mM glycine buffer at pH 8.9 and incubated for 3 h. The fluorescence intensity was measured at Ex/Em = 440 nm/484 nm using a SpectraMax iD3 Multi-Mode Microplate Reader (Molecular Devices).

### 2.7 Cell Viability Assay

HT22 cells were seeded in a 96-well microplate (SPL Life Sciences, Pocheon, Korea) at a density of 5 × 10^3^ cells/well. After 24 h of incubation, varying doses of the five TOMs at 10, 100, and 1,000 μg/mL were administered with monomeric tau K18 to measure cell viability against tau monomer-induced toxicity. After 48 h, a water-soluble tetrazolium 1 (WST-1) solution (DoGenBio, Seoul, Korea) was added to each well, and the cells were maintained in an incubator. To measure cell viability against tau aggregate-induced toxicity, tau aggregates were performed for 24 h. The tau aggregates were then added with the five TOMs at 10, 100, and 1,000 μg/mL to the HT22 cells. After 24 h, WST-1 solution was added to each well, and the cells were incubated. The cells were agitated for 1 min on a shaker, and the absorbance was measured at 450 nm using a SpectraMax iD3 multi-mode microplate reader (Molecular Devices).

### 2.8 Quantitative Reverse Transcription PCR (qRT-PCR) Analysis of Pro-inflammatory Cytokines

BV2 microglial cells were stimulated with 10 μM monomeric tau K18 or preformed tau K18 aggregates with or without treatment with the five TOMs for 6 h. Total RNA from BV2 cells was isolated using the AccuPrep RNA Extraction kit (Bioneer, Daejeon, Korea) according to the manufacturer's instructions and reverse-transcribed using TaKaRa PrimeScript RT Master Mix (Takara, Shiga, Japan). qRT-PCR was performed using iQ™ SYBR^®^ Green Supermix (Bio-Rad, CA, USA) to determine the expression of pro-inflammatory cytokines. To confirm the expression of cytokines in BV2 cells, the resultant cDNA was amplified using primers specific for TNF-α (sense:5’-TCG TAG CAA ACC ACC AAG TG-3’ and antisense:5’-ATA TAG CAA ATC GGC TGA CG-3’), IL-6 (sense:5’-GAG GAT ACC ACT CCC AAC AGA CC-3’ and antisense 5’-AAG TGC ATC ATC GTT GTT CAT ACA-3’) and GAPDH (sense:5’-TGG CAC AGT CAA GGC TGA GA-3’ and antisense:5’-CTT CTG AGT GGC AGT GAT GG-3’). The thermal cycling profile was as follows: 95°C for 3 min, 40 cycles of 95°C for 15 s, 59°C for 30 s, 72°C for 30 s, and 72°C for 10 min. The ∆∆Cq method with GAPDH was used to calculate the relative expression of each gene. The quantification cycle (Cq) values were normalized with GAPDH Cq and analyzed using the Comparative CT Method 2−∆∆Cq method.

### 2.9 Statistical Analysis

All the analyses were performed in a blinded manner for each group. Statistical analyses were conducted using the GraphPad Prism 9.0 software (GraphPad Software, Inc., CA, USA). Data are shown as mean ± standard error of the mean. The significance of the differences between the values for the two groups was analyzed using an independent t-test. The significance of differences between values for the three groups was analyzed using one-way analysis of variance with Fisher’s post-hoc test. ^#^*p* < 0.05 and ^###^*p* < 0.001 versus the control group. *p < 0.05, **p < 0.01, and ***p < 0.001 versus the vehicle group.

## 3 RESULTS

### 3.1 Inhibitory Effect of TOMs on Tau Hyperphosphorylation

**Fig (1):**
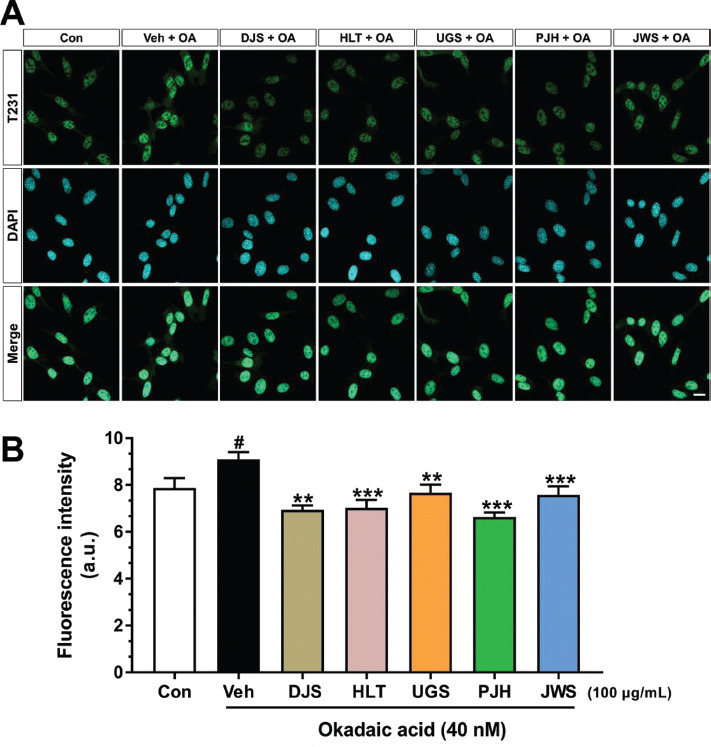
Effect of the five TOMs on okadaic acid-induced tau hyperphosphorylation. HT22 cells were co-treated with 40 nM okadaic acid and the five TOMs at the dose of 100 μg/ml for 4 h. (**A**) Representative image of the T231 staining in HT22 cells. (**B**) The fluorescence intensity of the p-tau^T231^ was remarkably decreased in the five TOMs-treated HT22 cells compared with vehicle-treated HT22 cells. Values are expressed as the mean ± S.E.M. Scale bar = 20 μm. ^#^*p* < 0.05 indicates significant differences compared with the control group (white bar). **p < 0.01 and ***p < 0.001 indicate significant differences compared with the vehicle-treated group (black bar). **Abbreviations: **Dangguijakyaksan; DJS, hwanglyeonhaedoktang; HLT, ukgansan; UGS, palmijihwanghwan; PJH, jowiseungchungtang; JWS, and okadaic acid; OA.

Tau hyperphosphorylation is the most crucial event in tau pathology. In particular, threonine (T) 231 is well-known to be the first residue to be phosphorylated in tau protein [53]. Moreover, the T231 residue is highly phosphorylated in the AD brain [54]. Thus, we induced the phosphorylation of the T231 residue of tau using okadaic acid [55, 56]. To examine the effects of the five TOMs on tau hyperphosphorylation, we performed ICC using an anti-phospho-tau (T231) antibody in HT22 cells treated with okadaic acid (Fig. **1A**). Quantitative results showed that the fluorescence intensity of p-tau^T231^ in vehicle-treated HT22 cells with okadaic acid was significantly increased than that in vehicle-treated HT22 cells without okadaic acid. In contrast, the fluorescence intensity of p-tau^T231^ in DJS-, HLT-, UGS-, PJH-, and JWS-treated HT22 cells with okadaic acid was remarkably decreased compared to that in vehicle-treated HT22 cells with okadaic acid (Fig. **1B**). Our results demonstrated that DJS, HLT, UGS, PJH, and JWS inhibited the phosphorylation of T231 residue induced by okadaic acid in HT22 mouse hippocampal neuronal cells.

### 3.2 Anti- and Dis-aggregation Effect of TOMs on Tau K18 Aggregation

**Fig (2):**
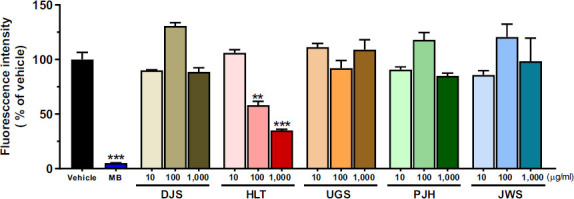
The inhibitory effects of aggregation from TOM on tau K18 monomer by assessing the fluorescence intensity of ThT. 100 μM MB and the five TOMs at doses of 10, 100, and 1000 μg/ml were incubated for 24 h with 35 μM tau K18 monomer. MB was used as a positive control. HLT at concentrations of 100 and 1,000 μg/ml significantly inhibits aggregation of tau K18 monomer. Values are expressed as the mean ± S.E.M. **p < 0.01 and ***p < 0.001 indicate significant differences compared with the vehicle-treated group (black bar). **Abbreviations: ** Methylene blue; MB, dangguijakyaksan; DJS, hwanglyeonhaedoktang; HLT, ukgansan; UGS, palmijihwanghwan; PJH, and jowiseungchungtang; JWS.

Hyperphosphorylated tau detaches from microtubules [57] and aggregates to form neurotoxic soluble aggregates [58]. Thus, we used heparin to aggregate monomeric tau K18 [59]. First, to investigate the effect of the five TOMs on tau aggregation, we performed a ThT assay using tau K18 fragments. The results showed that 100 μM MB significantly decreased ThT fluorescence intensity compared with that in the vehicle group. Similarly, 100 and 1,000 μg/ml of HLT significantly reduced ThT fluorescence intensity, compared with that in the vehicle group, indicating that HLT effectively inhibited the aggregation of monomeric tau K18 (Fig. **2**). However, DJS, UGS, PJH, and JWS did not show an inhibitory effect on tau aggregation at all concentrations.

**Fig (3):**
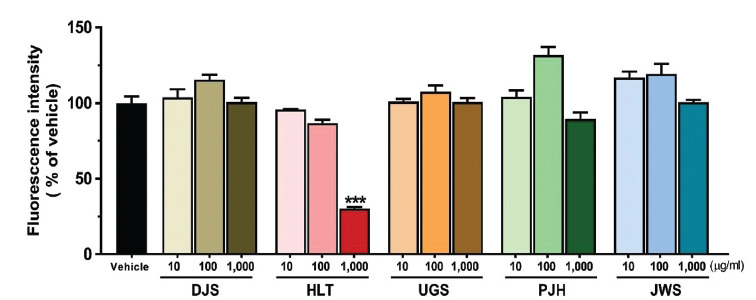
Effects of the five TOMs on dis-aggregation of tau K18 aggregate by assessing the fluorescence intensity of ThT in the presence and absence of the five TOMs. The five TOMs at doses of 10, 100, and 1000 μg/ml were incubated for 24 h with 35 μM tau K18 aggregate. HLT at a concentration of 1,000 μg/ml significantly increases the dis-aggregation of tau K18 aggregate. Values are expressed as the mean ± S.E.M. ***p < 0.001 indicates significant differences compared with the vehicle-treated group (black bar). **Abbreviations: ** Dangguijakyaksan; DJS, hwanglyeonhaedoktang; HLT, ukgansan; UGS, palmijihwanghwan; PJH, and jowiseungchungtang; JWS.

Next, to investigate the effect of the five TOMs on mature tau aggregates, we formed tau aggregates by incubating tau K18 fragments for 24 h. The formed tau aggregates were treated with five TOMs and incubated for 21 h. ThT fluorescence intensity was significantly reduced in cells treated with 1,000 μg/ml HLT compared with that in the vehicle group, indicating that high concentrations of HLT were effective in promoting the dissociation of tau K18 aggregates. However, DJS, UGS, PJH, and JWS did not affect the dissociation of oligomeric tau K18 (Fig. **3**). These results showed that only HLT inhibited tau aggregation and promoted the degradation of tau aggregates.

### 3.3 Neuroprotective Effect of TOMs on Tau K18-induced Neurotoxicity

**Fig (4):**
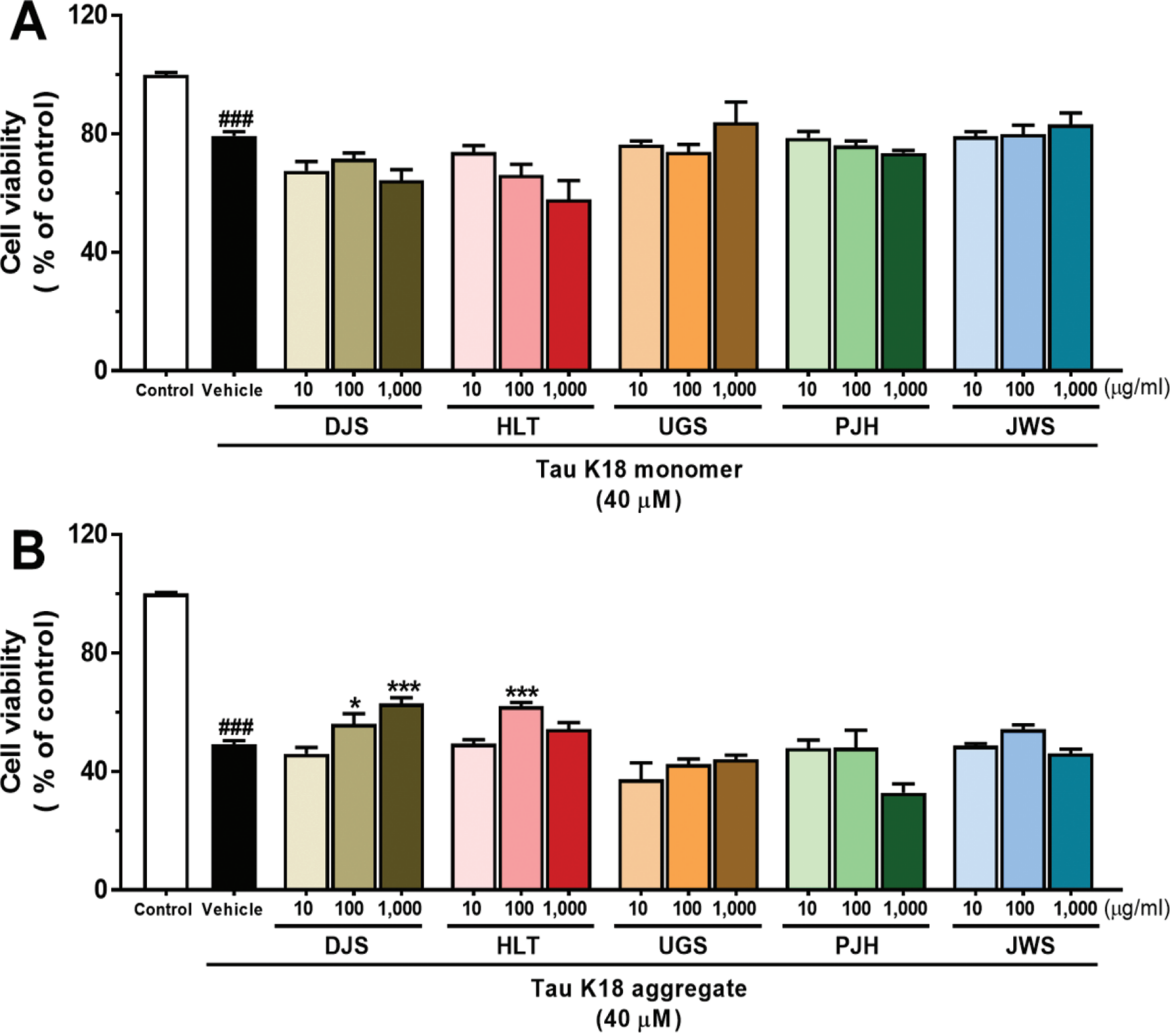
The protective effects of the five TOMs on tau K18-induced neurotoxicity. The HT22 cells were co-treated with 40 μM monomer (**A**) and oligomer (**B**) of tau K18 and the five TOMs at doses of 10, 100, and 1000 μg/ml for 48 h or 24 h, individually. Cell viability was assessed by WST-1 assay. DJS at concentrations of 100 and 1,000 μg/ml and HLT at 100 μg/ml treatment significantly ameliorate tau K18 oligomer-induced neuronal loss. Values are expressed as the mean ± S.E.M. ^###^*p* < 0.001 indicates significant differences compared with the control group (white bar). *p < 0.05 and ***p < 0.001 indicate significant differences compared with the vehicle-treated group (black bar). **Abbreviations: ** Dangguijakyaksan; DJS, hwanglyeonhaedoktang; HLT, ukgansan; UGS, palmijihwanghwan; PJH, and jowiseungchungtang; JWS.

The oligomeric species of tau is the most neurotoxic form of the other tau species, and these tau aggregates induce neurodegeneration [60, 61]. To determine whether the five TOMs have neuroprotective effects against tau-induced neurotoxicity, we confirmed the effect of neuroprotection *via * the WST-1 assay in HT22 hippocampal neuronal cells. We measured cell viability under two conditions. The HT22 cells were treated with 40 μM tau monomer and vehicle or various concentrations of the five TOMs. Cell viability was significantly decreased in tau-provoked HT22 cells treated with a vehicle, compared with the control. The five TOMS did not show a neuroprotective effect against tau-induced neurotoxicity at all concentrations. (Fig. **4A**). Next, the cells were treated with 40 μM tau aggregates and vehicle or various concentrations of the five TOMs. Cell viability was dramatically reduced in vehicle-treated HT22 cells stimulated with tau aggregates, compared with that observed in the control.

Interestingly, neuronal cell death induced by oligomeric tau was more severe than neuronal loss induced by monomeric tau in HT22 cells. Upon stimulation by tau aggregates, the viability of HT22 cells treated with 100 and 1,000 μg/ml of DJS and 100 μg/ml of HLT significantly increased, compared with that of HT22 cells treated with the vehicle. However, UGS, PJH, and JWS did not show neuroprotective effects against tau aggregate-induced neurodegeneration at any concentration (Fig. **4B**). Our findings indicate that DJS and HLT have neuroprotective effects against neuronal cell death induced by tau aggregates.

### 3.4 Anti-inflammatory Effect of TOMs on Tau K18-induced Neuroinflammation

**Fig (5):**
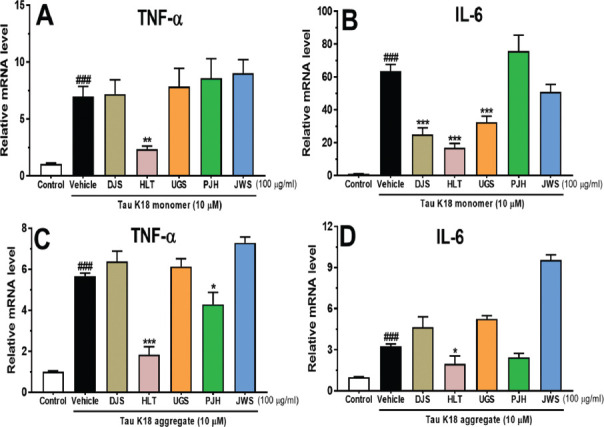
The attenuating effects of the five TOMs on tau K18-induced neuroinflammation. The BV2 cells were co-treated with 10 μM monomer (**A**, **B**) and aggregate (**C**, **D**) of tau K18 and the five TOMs at 100 μg/ml for 6 h. Expression levels of TNF-α (**A**, **C**) and IL-6 (**B**, **D**) were measured by qRT-PCR. HLT significantly inhibited the production of TNF-α in tau monomer or aggregate-treated BV2 cells. PJH significantly suppressed the release of TNF-α in tau aggregate-treated BV2 cells. Furthermore, DJS, HLT, and UGS significantly inhibited the production of IL-6 in tau monomer-treated BV2 cells. In addition, HLT significantly suppressed the release of IL-6 levels in tau aggregate-treated BV2 cells. Values are expressed as the mean ± S.E.M from triplicate experiments. ^###^*p* < 0.001 indicates significant differences compared with the control group (white bar). *p < 0.05, **p < 0.01, and ***p < 0.001 indicate significant differences compared with the vehicle-treated group (black bar). **Abbreviations: ** Dangguijakyaksan; DJS, hwanglyeonhaedoktang; HLT, ukgansan; UGS, palmijihwanghwan; PJH, and jowiseungchungtang; JWS.

The aggregates of hyperphosphorylated tau upregulate pro-inflammatory cytokines, such as TNF-α and IL-6 [62, 63]. To evaluate the effects of the five TOMs on the tau-induced neuroinflammatory response, we measured the mRNA levels of pro-inflammatory cytokines in BV2 microglial cells using qRT-PCR. BV2 cells treated with tau K18 monomer for 6 h exhibited increased TNF-α and IL-6 levels compared with control cells not treated with tau K18 (Figs. **5A**, **B**). Interestingly, HLT significantly inhibited the production of TNF-α in tau monomer-treated BV2 cells (Fig. **5A**). Furthermore, treatment with DJS, HLT, and UGS significantly suppressed IL-6 levels in tau monomer-treated BV2 cells. Next, BV2 cells treated with tau K18 aggregate for 6 h exhibited increased TNF-α and IL-6 levels compared with the control cells not treated with tau K18 aggregate (Figs. **5C**, **D**). Notably, HLT and PJH significantly inhibited the release of TNF-α in tau aggregate-treated BV2 cells (Fig. **5C**). Moreover, treatment with HLT significantly suppressed IL-6 levels in tau aggregate-treated BV2 cells (Fig. **5D**). These results suggest that DJS, HLT, UGS, and PJH may inhibit tau-induced production of neuroinflammatory cytokines.

## 4 DISCUSSION

**Fig. (6):**
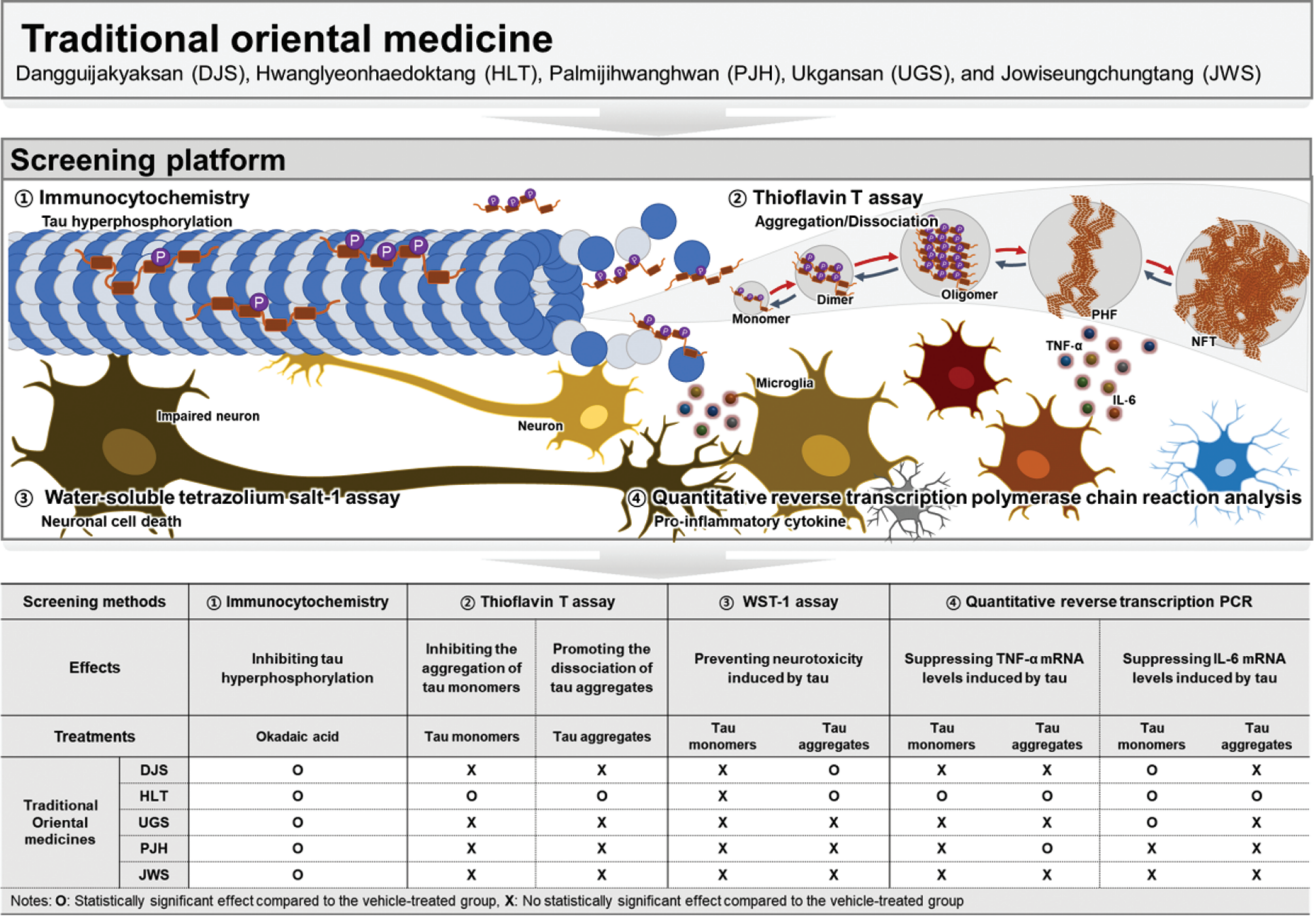
Graphical representation of traditional orientation medicine.

TOMs have been utilized for a considerable amount of time in East Asian nations to alleviate the symptoms of dementia, including AD. Among the diverse types of TOMs, DJS, HLT, UGS, PJH, and JWS are the most commonly used agents for the treatment of AD-related symptoms, such as cognitive declines [32, 64]. However, only a few studies have reported the direct effects of these five TOMs on AD-related pathology, particularly tau pathology. In this study, to investigate whether five TOMs can alleviate tau pathology, we assessed the effect of the five TOMs using cell-free and cell-based screening approaches. First, we evaluated the effects of the five TOMs on hyperphosphorylated tau using ICC. The five TOMs inhibited okadaic acid-induced hyperphosphorylation of tau in HT22 cells (Fig. **1**). Next, we assessed the effects of the five TOMs on tau aggregation and dissociation using the ThT assay (Figs. **2**, **3**). Only HLT directly inhibited the aggregation of tau and promoted the degradation of tau aggregates. Subsequently, we confirmed the effects of the five TOMs against neurotoxicity induced by tau using the WST-1 assay, and we found that DJS and HLT inhibited neuronal cell death induced by tau aggregates in HT22 cells (Fig. **4**). Finally, we examined the effects of the five TOMs on pro-inflammatory cytokines using qRT-PCR. DJS, HLT, UGS, and PJH downregulated the mRNA levels of TNF-α and IL-6, which were upregulated by tau in BV2 cells (Fig. **5**). Taken together, our findings demonstrate that the five TOMs have beneficial effects on tau pathology (Fig. **6**).

We found that HLT inhibited hyperphosphorylated tau, aggregation and degradation of tau, and tau-induced pro-inflammatory cytokines. However, no significant inhibition of monomeric tau-induced cytotoxicity was observed. The absence of neuroprotection of HLT against monomeric tau-induced cytotoxicity is likely due to the cytotoxicity observed in HLT at a concentration of 50 μg/ml (Fig. **S2**). HLT induces cell arrest by inactivating phospho-Cdc2 and phospho-Cdc25C and reducing proteins such as cyclin A and cyclin B1 [65]. In addition, baicalein, a component of HLT, inhibits the phosphorylation of IkB-α, which induces the activity of caspase-9 and caspase-3 and consequently inhibits cell proliferation [66]. Despite the significant effect of HLT on tau pathology, its lack of neuroprotective effect is likely attributable to its ability to induce apoptosis and inhibit proliferation. Therefore, the control of tau pathology using HLT requires the administration of optimal concentrations that will not cause adverse effects.

Although DJS did not directly affect tau aggregation and degradation, it significantly inhibited neurotoxicity induced by tau aggregate. Notably, the six medicinal herbs, *Atractylodes lancea* (AL), *Poria*, Peony, *Alisma*, *Cnidium*, and *Angelica* in DJS, have neuroprotective effects. AL has diverse pharmacological properties, including neuroprotection [67]. *Poria* showed a neuroprotective effect through the inhibition of the MAPK/NF-κB pathway in the hippocampus of a rat model of AD induced by D-galactose and aluminum trichloride [68]. Peony and *Alisma* have the ability to protect cells against oxidative stress [69, 70]. *Cnidium* has been shown to inhibit neurotoxic and oxidative damage in cortical neurons in an ischemia rat model [71]. It is well known that the crude extracts and isolated components of JAR present in DJS exhibit neuroprotective and cognitive-enhancing effects [72]. In particular, *Angelica* inhibits Aβ-induced neurotoxicity and rescues Aβ-induced memory impairment in AD models [73-75]. Therefore, the six medicinal herbs that are present in DJS can inhibit neurotoxicity caused by tau aggregates.

A previous review [32] addressed the commonly used TOMs in the clinical treatment of patients with cognitive impairment, including AD. In particular, DJS, HLT, and PJH have been reported to exert beneficial effects on AD. DJS, also known as *danggui Shaoyao San* in Chinese and *tokishakuyakusan* in Japan, consists of several herbs, including *Angelica sinensis* (AS), *Paeonia lactiflora*, and *Ligusticum chuanxiong*, which ameliorate AD pathology. DJS ameliorates cognitive impairment by regulating Akt/GSK3β/β-catenin signaling and hippocampal estrogen synthesis [76, 77]. AS prevents the hyperphosphorylation of tau by regulating the PI3K/Akt/GSK3 signaling pathway and protects the brain from Aβ-induced neurotoxicity and neuroinflammation [78-80]. Moreover, paeoniflorin can inhibit tau hyperphosphorylation by reducing inflammatory cytokine production and regulating the SOCS2/IRS-1 and calpain/Akt/GSK3β pathways [81, 82]. HLT consists of *Coptis chinensis* (CC), *Scutellaria baicalensis* (SB), *Phellodendron amurense*, and *Gardenia jasminoides*. Berberine extracted from CC suppresses the hyperphosphorylation of APP and tau *via* the Akt/GSK3 signaling pathway [83]. WG, another component of SB, ameliorates cognitive decline in 3xTg-AD mice and inhibits aggregation of Aβ and hyperphosphorylation of tau [47]. PJH is an herbal composition consisting of eight herbs that have been reported to exert numerous therapeutic effects on AD [84-89]. Among the individual herbs, *catalpol* has been found to alleviate memory loss by reducing Aβ levels and regulating enzymes associated with reactive oxygen species [87]. Another active compound, 1,2,3,4,6-penta-O-galloyl-β-D-glucopyranose, inhibits the aggregation of Aβ and dissociates Aβ aggregates [85]. Previous studies have already demonstrated the effect of JWS and UGS on AD pathology [36, 45, 46]. In particular, some studies demonstrated the alleviating effect of UR, a constituent of UGS, on Aβ- and tau-induced neuronal loss and neuroinflammation, along with its dual regulatory effect, involving both the anti- and dis-aggregation of Aβ and tau aggregates [36, 46]. Therefore, using molecular docking simulations, the molecular mechanism underlying the effect of UR on Aβ and tau aggregation/dissociation was analyzed to determine whether UR alleviates Aβ and tau accumulation by directly interacting with Aβ and tau. Surprisingly, rhynchophylline and corynoxeine, the bioactive components of *Uncaria rhynchophylla*, bind to the aggregation-related domains of Aβ and tau. This binding significantly inhibits tau aggregation and promotes the dissociation of Aβ and tau aggregates.

Identifying new compounds that can target different tau pathologies is an important therapeutic strategy for AD. Therefore, it is important to investigate the effects of TOMs on tau pathology. However, we have not yet conducted experiments to examine the effects of TOMs in animal models of AD. Therefore, in further studies, we aim to evaluate the *in vivo* efficacy of the five TOMs in animal models that exhibit tau-related pathologies, such as 3xTg and P301S transgenic mice. Furthermore, future experiments are needed to further characterize the five TOMs across various tau pathologies, including tau protein kinase, tau propagation, and microtubule stabilization, and identify the principal ingredients that modulate the aggregation and dissociation of tau.

## CONCLUSION

In conclusion, we revealed that the five TOMs inhibited tau hyperphosphorylation in HT22 hippocampal neuronal cells. In addition, only HLT suppressed the aggregation of tau K18 and promoted the dissociation of tau K18 aggregates. Moreover, DJS and HLT inhibited neurotoxicity caused by tau aggregates in HT22 cells. Furthermore, DJS, HLT, UGS, and PJH downregulated tau-induced neuroinflammatory responses in BV2 cells. Collectively, HLT showed the greatest efficacy among the five TOMs in cell-free and cell-based screening approaches. Lastly, these findings suggest that HLT is a suitable therapeutic agent for AD patients with tau pathology.

## AUTHORS’ CONTRIBUTIONS

All authors contributed to this present work: Conceptualization was done by M.M.; methodology was presented by S.K., Y.N., and S.J.S.; supervision was provided by S.K., Y.N., S.J.S., and M.M.; project administration was contributed by S.K., Y.N., S.J.S., and M.M.; investigation was done by H.H.P., B-H.K., S.H.L., Y.H.P., and H.C.; visualization was done by H.H.P., B-H.K., S.H.L., Y.H.P., and H.C.; data curation was conducted by S.K., Y.N., S.J.S., and M.M.; validation was provided by S.K., Y.N., S.J.S., and I.P.; formal analysis was performed by H.H.P., B-H.K., S.H.L., Y.H.P., and D-H.Y.; original draft was written by H.H.P., B-H.K., S.H.L., and Y.H.P.; review and editing were performed by S.K., Y.N., S.J.S., and M.M. All authors read and agreed to the published version of the manuscript.

## Data Availability

The authors confirm that the data supporting the findings of this research are available within the article and its supplementary materials.

## LIST OF ABBREVIATIONS

AD: Alzheimer's Disease
Aβ: Amyloid-β
AL: *Atractylodes lancea*
ALR: *Atractylodes lancea* Rhizome
AR: *Alisma* Rhizome
AS: *Angelica sinensis*
BSA: Bovine Serum Albumin
CC: *Coptis chinensis*
CR: *Cnidium* Rhizome
DJS: Dangguijakyaksan
DMEM: Dulbecco’s Modified Eagle’s Medium
DMEM/F12: Dulbecco’s Modified Eagle’s Medium/F12 Nutrient Mixture Ham
DPBS: Dulbecco's Phosphate-buffered Saline
HLT: Hwanglyeonhaedoktang
ICC: Immunocytochemistry
IL-6: Interleukin-6
JAR: Japanese *Angelica* Root
JWS: Jowiseungchungtang
MB: Methylene Blue
NFTs: Neurofibrillary Tangles
PBS: Phosphate-buffered Saline
PHF: Paired Helical Filaments
PJH: Palmijihwanghwan
PS: *Poria* Sclerotium
qPCR: Quantitative Polymerase Chain Reaction
SB: *Scutellaria baicalensis*
ThT: Thioflavin T
TOMs: Traditional Oriental Medicines
TNF-α: Tumor Necrosis Factor-α
UGS: Ukgansan
UR: *Uncaria rhynchophylla*
WG: Wogonin
WST-1: Water-soluble Tetrazolium 1
