# Supplementary material for: Therapeutic Potential of Traditional Oriental Medicines in Targeting Tau Pathology: Insights from Cell-free and Cell-based Screening
Source: Curr Med Chem. 2024 Mar 13;32(9):1830–45. doi: 10.2174/0109298673295901240311072440 (PMC12246740; doi:10.2174/0109298673295901240311072440)
Supplement: Supplementary file 1 [file CMC-32-9-1830_SD1.pdf]

Supplementary Material

Therapeutic Potential of Traditional Oriental Medicines in Targeting Tau Pathology: Insights from Cell-free and Cell-based Screening

Hyun Ha Park[1,#], Byeong-Hyeon Kim[1,#], Seol Hwa Leem[1,#], Yong Ho Park[1,#], Hyunju Chung[2], Doo-Han Yoo[3,4], Insu Park[5], Yunkwon Nam[1,*], Sujin Kim[1,3,*], Soo Jung Shin[1,*] and Minho Moon[1,3,*]

[1]Department of Biochemistry, College of Medicine, Konyang University, 158, Gwanjeodong-ro, Seo-gu, Daejeon, 35365, Republic of Korea; [2]Department of Core Research Laboratory, Medical Science Research Institute, Kyung Hee University Hospital Gangdong, Seoul, 05278, Republic of Korea; [3]Research Institute for Dementia Science, Konyang University, 158, Gwanjeodong-ro Seo-gu, Daejeon, 35365, Republic of Korea; [4]Department of Occupational Therapy, Konyang University, 158, Gwanjeodong-ro, Seo-gu, Daejeon, 35365, Republic of Korea; [5]Department of Biomedical Engineering, Konyang University, 158, Gwanjeodong-ro, Seo-gu, Daejeon, 35365, Republic of Korea

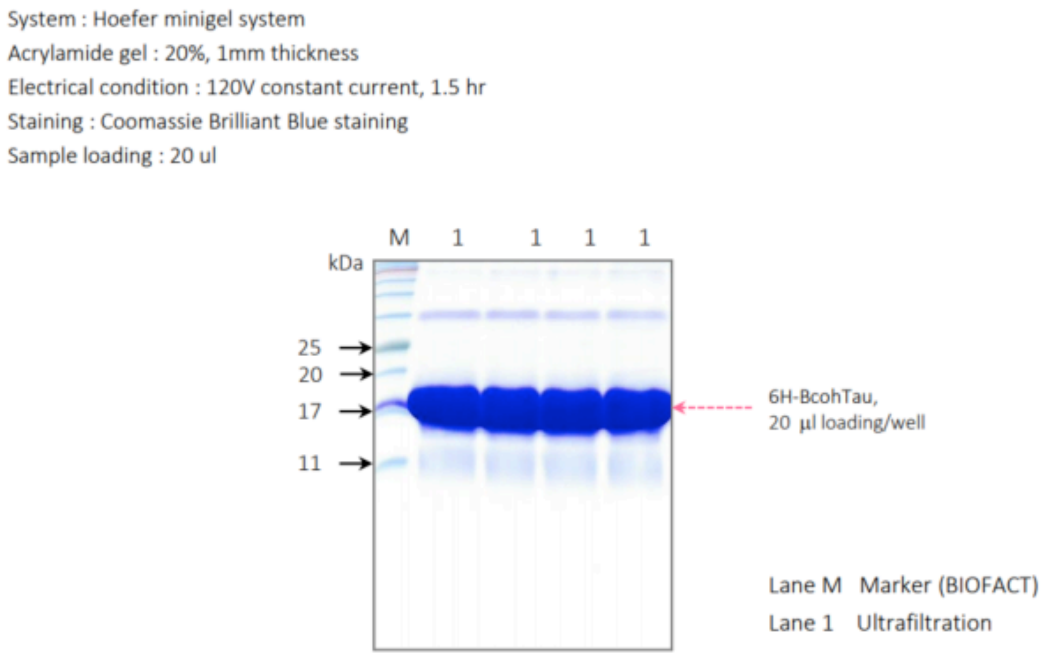

Supplementary Fig. (1). Recombinant tau K18 purification. Sodium dodecyl sulfate-polyacrylamide gel electrophoresis was used to validate the presence of the recombinant tau K18. The remaining lanes contain purified protein, whereas the marker represents the protein's molecular weight.

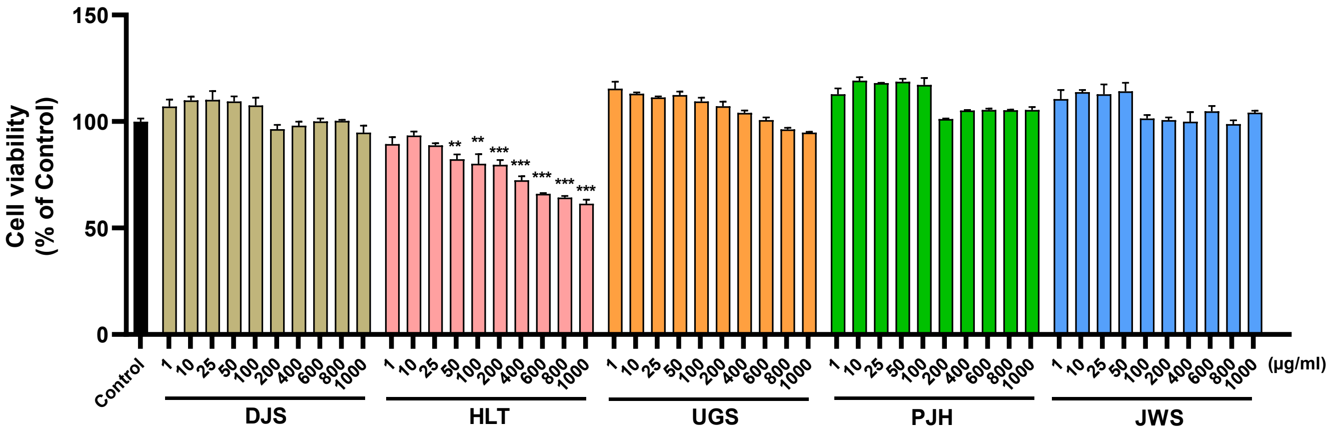

Supplementary Fig. (2). Cell viability of five TOMs in HT22 cells. The cytotoxicity of HLT was observed from 50 to 1,000 μg/ml compared to the control group. DJS, UGS, PJH, and JWS did not show cytotoxicity in mouse hippocampal neuronal cells. Values are expressed as the mean ± S.E.M. $^{**}p < 0.01$ and $^{***}p < 0.001$ indicate significant differences compared with the control group (black bar). Dangguijakyaksan; DJS, hwanglyeonhaedoktang; HLT, ukgansan; UGS, palmijihwanghwan; PJH, and jowiseungchungtang; JWS.
